# An Uncommon Fracture: A Case Report of a Scapular Fracture in a Young Patient

**DOI:** 10.7759/cureus.76414

**Published:** 2024-12-26

**Authors:** Daniela Saenz, Marcela Palomo, Javier Ardebol

**Affiliations:** 1 Medical Research, Universidad Francisco Marroquín, Guatemala, GTM

**Keywords:** fracture, injury, scapula, shoulder, trauma

## Abstract

Scapular fractures are rare, typically resulting from high-energy trauma. These injuries frequently present alongside other thoracic or shoulder injuries. Patients often exhibit posterior shoulder pain, swelling, and limited range of motion, which can suggest a variety of shoulder pathologies.

This case presents a scapular fracture in a 32-year-old male patient following a high-impact accident. The patient presented with posterior shoulder pain and restricted movement, initially raising suspicion of a complex shoulder injury. This report underscores the importance of considering scapular fractures in patients with these nonspecific symptoms and highlights the role of detailed imaging for diagnosis. Early recognition and appropriate classification of scapular fractures guide treatment, minimize complications, and promote optimal functional recovery, emphasizing the need for awareness among clinicians.

## Introduction

Scapular fractures are rare injuries, accounting for only 0.5%-1% of all fractures. Their rarity can be attributed to the scapula's protected anatomical position, where it is shielded by the ribcage and surrounded by dense musculature, requiring substantial force from high-energy trauma to result in a fracture. Such trauma typically includes incidents like motor vehicle collisions or significant falls [1]. The scapula’s intricate structure, comprising the body, glenoid cavity, acromion, and coracoid process, makes fracture classification essential for precise diagnosis and effective treatment.

Fractures of the scapular body are the most common and are often treated nonoperatively with favorable outcomes. In contrast, fractures involving the glenoid cavity, particularly intra-articular ones, demand careful evaluation due to their potential to compromise shoulder joint stability and function [2]. Imaging is pivotal in diagnosing scapular fractures; while radiographs provide an initial overview, computed tomography (CT) is invaluable for assessing complex fractures and joint involvement [2,3].

This case report examines a scapular fracture, emphasizing the significance of imaging in diagnosis, fracture classification, and the tailored approaches required for treatment, while addressing the challenges inherent in managing such uncommon injuries.

## Case presentation

A 32-year-old male patient presented to the emergency department with left shoulder pain following a motorcycle accident in which he collided with a truck. His past medical history was unremarkable except for an appendectomy performed two years earlier. Physical examination of the left shoulder revealed no visible wounds, bruising, or abrasions but demonstrated a mild deformity of the left scapular region, characterized by subtle asymmetry of the shoulder contour and localized swelling. Both passive and active ranges of motion were limited by pain. Clinical tests, including Apley and Jobe tests, were deferred due to pain. Sensation was intact locally and distally, with no evidence of proximal or distal nerve injury. The cardiopulmonary assessment was normal.

**Figure 1:**
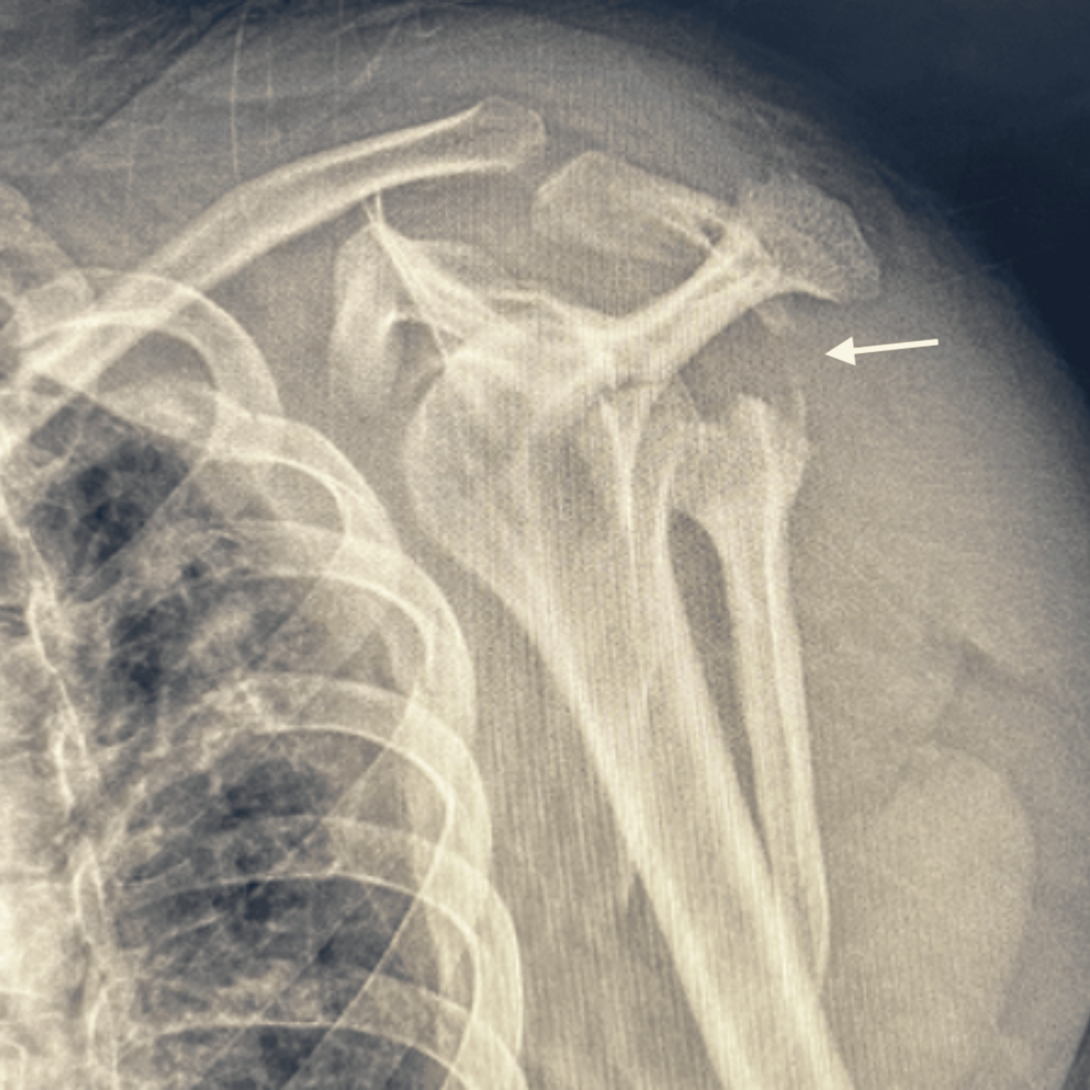
Scapular Y-radiographic view of the left shoulder showing a multifragmentary fracture.

**Figure 2:**
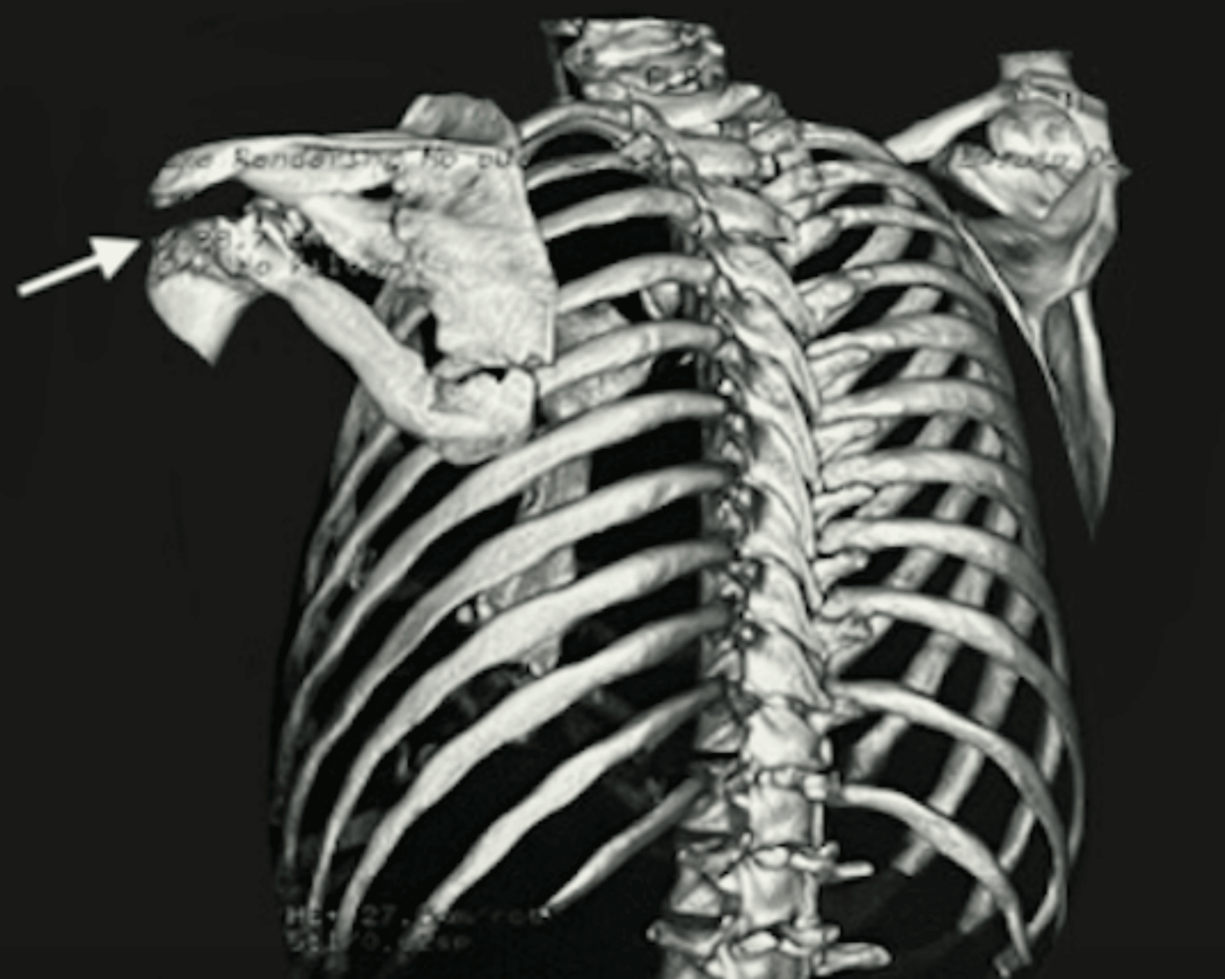
Thoracic CT bone reconstruction with evidence of multifragmentary fracture involving both the scapular body and the glenoid cavity. CT: computed tomography

**Figure 3:**
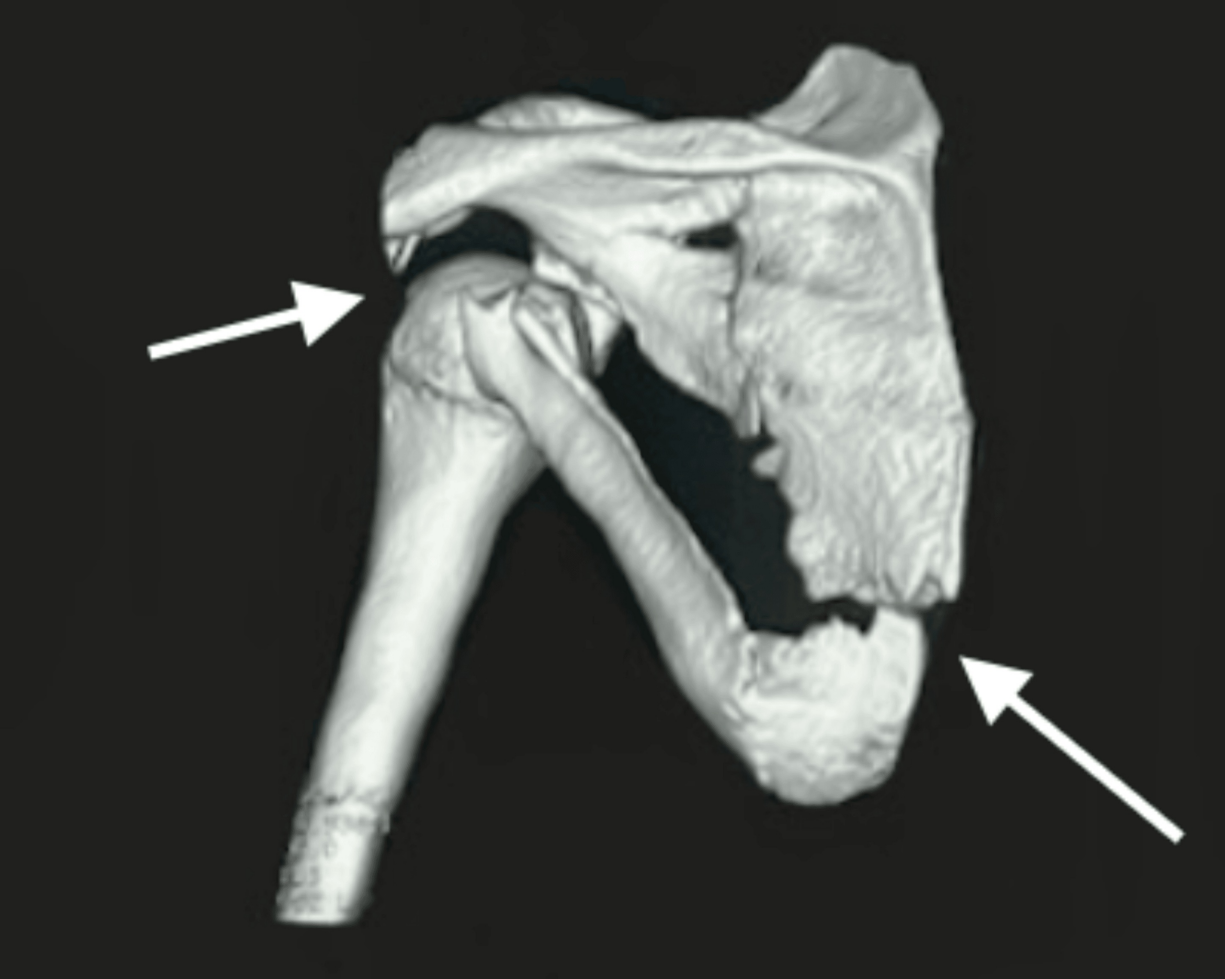
Left shoulder CT bone reconstruction with evidence of multifragmentary fracture involving both the scapular body and the glenoid cavity. CT: computed tomography

To further evaluate the injury, radiographs were obtained, revealing a multifragmentary fracture of the scapular body (Figure 1). Given these findings, further imaging was performed for a more thorough assessment. Three-dimensional CT reconstructions (Figures 2, 3) demonstrated a multifragmentary fracture involving both the scapular body and the glenoid cavity, with intra-articular involvement.

**Figure 4:**
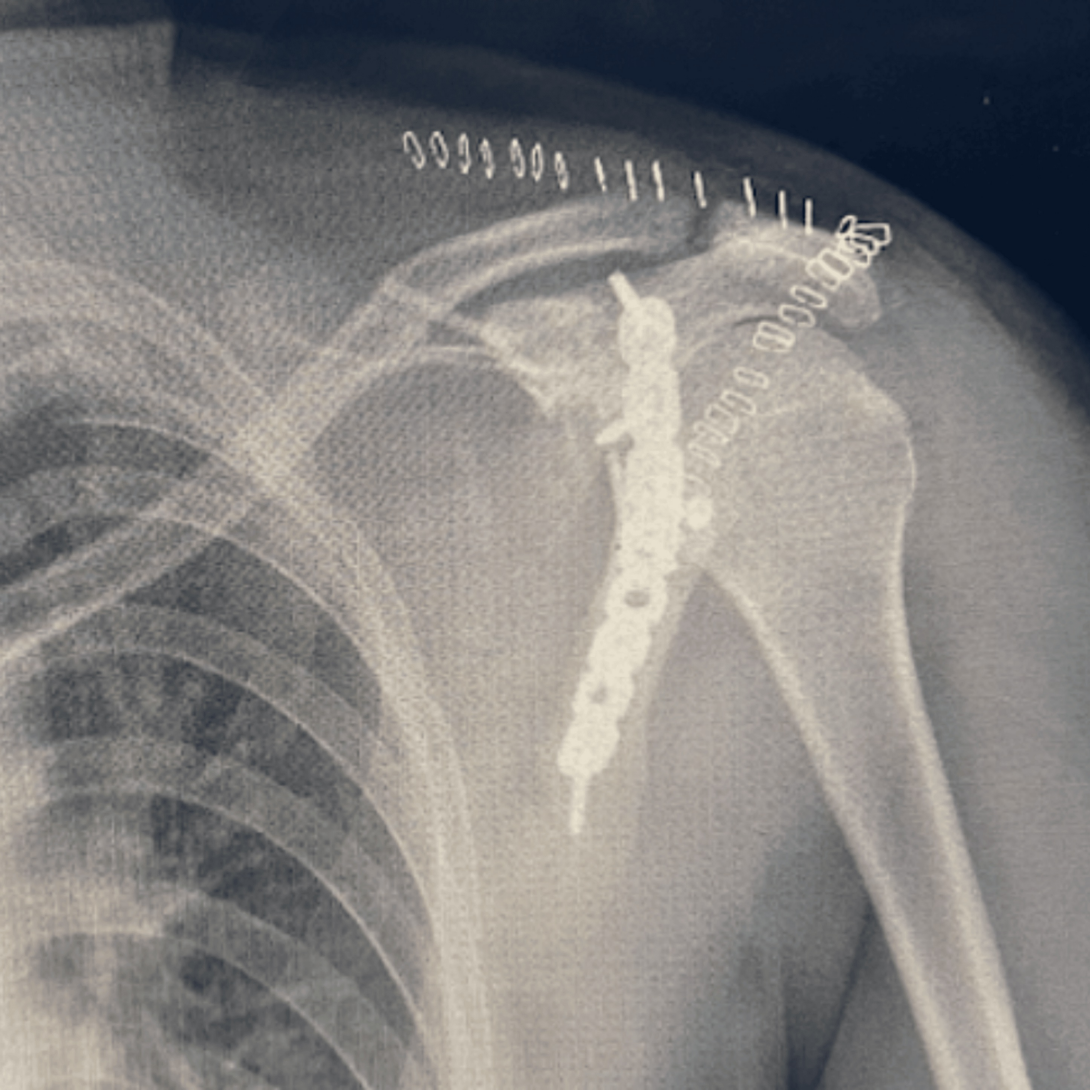
Postoperative AP radiographic view of the left shoulder.

**Figure 5:**
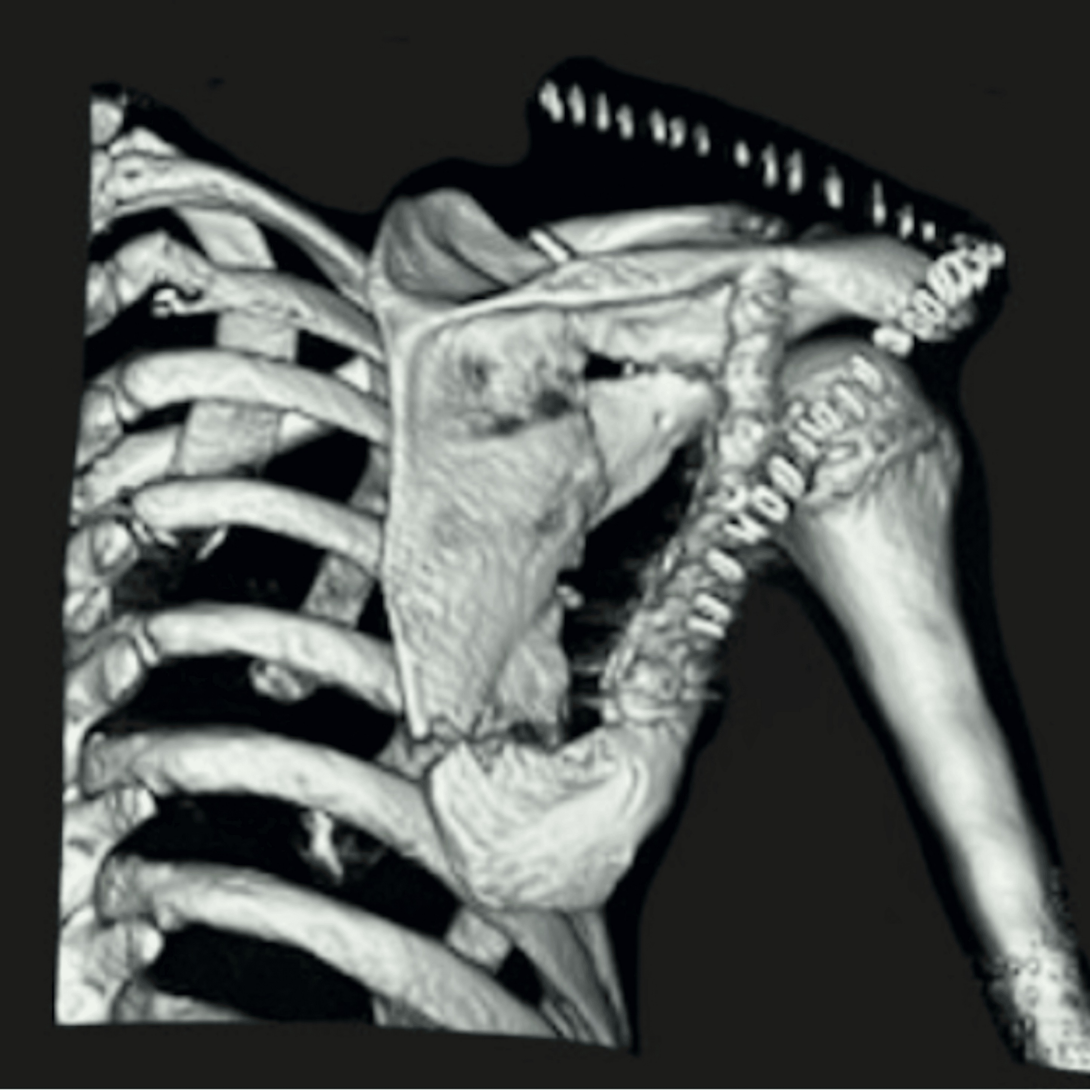
Postoperative CT bone reconstruction of the left shoulder. CT: computed tomography

The patient underwent open reduction and internal fixation (ORIF) via the Judet approach under block anesthesia with sedation [4]. Partial dissection of the deltoid muscle was performed to access the fracture site through the rotator cuff muscles. The fracture was reduced, and a pre-contoured titanium plate was secured using proximal and distal cortical screws. The articular capsule was repaired using fiber wire and a hard anchor. Immediate postoperative imaging (Figures 4, 5) confirmed proper alignment and fixation.

At the one-week follow-up visit, the patient reported significant pain reduction but continued to experience mild discomfort with certain movements. Physical examination revealed a healing surgical site with no signs of infection, swelling, or drainage. At this visit, a rehabilitation protocol was provided, including passive range of motion exercises, and sling use was recommended until the four-week postoperative mark.

## Discussion

Scapular fractures, though infrequent compared to other skeletal injuries, present unique challenges in diagnosis, management, and rehabilitation. These injuries account for approximately 0.5% of all fractures, with an annual incidence of scapular fractures ranging from 0.5 to 1.0 fractures per 100,000 people [1]. More specifically, they represent only 1.6% to 7.3% of upper extremity fractures [1,2].

Scapular fractures are typically classified by both location and injury pattern, each with distinct implications for treatment and recovery. Location-based classifications include fractures of the scapular body, glenoid, acromion, and coracoid processes. While scapular body fractures account for about half of these injuries, glenoid fractures represent around 10% of scapular fractures but are more detrimental, as they are often linked to glenohumeral instability [3]. The Ideberg classification is commonly used for glenoid fractures, categorizing them into six types based on the pattern and extent of intra-articular involvement [5]. Type I fractures involve isolated glenoid rim fractures, Type II fractures are caused by fractures of the glenoid with associated dislocations of the humeral head, Type III fractures affect the entire glenoid, Type IV fractures involve the glenoid with associated fractures of the scapular neck, Type V fractures involve fractures with scapular body fractures, and Type VI fractures involve complex fractures with associated scapular body and acromion fractures. This classification is critical for determining the severity of joint disruption and guiding treatment strategies.

Scapular fractures are also categorized as intra-articular or extra-articular [5,6]. Intra-articular fractures, which involve joint surfaces (i.e., the glenoid cavity), present a higher risk of joint instability, chronic pain, and posttraumatic arthritis due to articular surface disruption [6]. In contrast, extra-articular fractures-such as those affecting the scapular body, acromion, and coracoid process-do not involve the joint itself and generally do not compromise stability [6]. Distinguishing between intra- and extra-articular fractures is crucial, as it shapes both immediate management and long-term shoulder function. Additionally, clinicians should be aware of scapulothoracic dissociation, a rare but life-threatening injury characterized by complete separation of the scapula from the thoracic cage [7]. Scapulothoracic dissociation has a very high association with neurovascular injury, including brachial plexus and vascular injury, and requires immediate surgical intervention. Diagnostic confirmation involves advanced imaging such as CT scans and MRI, and management often includes emergency stabilization and surgical reconstruction of the scapulothoracic articulation, along with addressing any associated neurovascular deficits [7].

Diagnosing scapular fractures requires a high index of suspicion, particularly when associated with other injuries, such as rib or lung trauma [8]. Clinical evaluation should include a thorough history and physical examination, focusing on assessing range of motion and any signs of associated soft tissue injuries. Radiographic imaging, including standard anteroposterior and lateral views, is essential. However, CT scans provide more detailed imaging of complex fractures, particularly in the glenoid region, where traditional X-rays lack specificity [9].

Management of scapular fractures depends on the fracture type, displacement, and associated injuries. Nondisplaced or minimally displaced fractures often respond well to conservative management, including analgesia, immobilization, and physical therapy [10]. The primary goal is to promote early mobilization while minimizing pain and maintaining shoulder function. In contrast, displaced or intra-articular fractures often require surgery. ORIF can stabilize complex fractures, particularly those involving the glenoid or acromion. Indications for surgery typically include significant displacement, instability, or a fracture leading to functional impairment [11].

Rehabilitation after scapular fractures is important for achieving optimal outcomes. The goals of rehabilitation include restoring shoulder mobility, strength, and function [12]. The timeline for rehabilitation varies; typically, patients can expect a return to normal activities within 12 weeks for nondisplaced fractures, while those requiring surgery usually have longer rehabilitation timelines [13]. Patient education regarding the importance of adhering to rehabilitation protocols is essential for successful recovery.

## Conclusions

This case report describes a rare, isolated scapular fracture. Prompt diagnosis and appropriate management are essential for optimal recovery and functional outcomes. Surgical intervention aims to restore shoulder stability and mobility, improving the patient's quality of life. A multidisciplinary approach-incorporating orthopedic, surgical, and rehabilitation expertise-is recommended to ensure the best possible results and minimize complications. Additionally, recognizing rare complications like scapulothoracic dissociation, associated with neurovascular injuries, is crucial for preventing long-term disability. Effective management requires accurate fracture classification, appropriate imaging, and a coordinated approach to achieve optimal patient outcomes.
